# Emo-FilM: A multimodal dataset for affective neuroscience using naturalistic stimuli

**DOI:** 10.1038/s41597-025-04803-5

**Published:** 2025-04-23

**Authors:** Elenor Morgenroth, Stefano Moia, Laura Vilaclara, Raphael Fournier, Michal Muszynski, Maria Ploumitsakou, Marina Almató-Bellavista, Patrik Vuilleumier, Dimitri Van De Ville

**Affiliations:** 1 Neuro-X Institute, École Polytechnique Fédérale de Lausanne, Geneva, 1202 Switzerland; 2 Department of Radiology and Medical Informatics, University of Geneva, Geneva, 1202 Switzerland; 3 Swiss Center for Affective Sciences, University of Geneva, Geneva, 1202 Switzerland; 4 Department of Basic Neurosciences, University of Geneva, Geneva, 1202 Switzerland; 5 CIBM Center for Biomedical Imaging, Geneva, 1202 Switzerland

**Keywords:** Emotion, Psychology

## Abstract

The Emo-FilM dataset stands for Emotion research using Films and fMRI in healthy participants. This dataset includes emotion annotations by 44 raters for 14 short films with a combined duration of over 2½ hours and recordings of respiration, heart rate, and functional magnetic resonance imaging (fMRI) from a sample of 30 individuals watching the same films. 50 items were annotated including discrete emotions and emotion components from the domains of appraisal, motivation, motor expression, physiological response, and feeling. The ratings had a mean inter-rater agreement of 0.38. The fMRI data acquired at 3 Tesla is includes high-resolution structural and resting state fMRI for each participant. Physiological recordings included heart rate, respiration, and electrodermal activity. This dataset is designed, but not limited, to studying the dynamic neural processes involved in emotion experience. It has a high temporal resolution of annotations, and includes validations of annotations by the fMRI sample. The Emo-FilM dataset is a treasure trove for researching emotion in response to naturalistic stimulation in a multimodal framework.

## Background & Summary

Neuroimaging under naturalistic conditions is a growing field within neuroscience, which has been proven useful in a variety of subjects including language^1,2^, social cognition^3^ and emotion^4,5^. While the nature of functional Magnetic Resonance Imaging (fMRI) intrinsically limits the observation of naturalistic conditions, movies and films can be easily implemented as naturalistic paradigms in the scanner. In particular, film fMRI opens a range of new pathways for understanding the brain, as reflected by an increasing push towards naturalistic and other non-traditional paradigms in the field^6^.

Film fMRI is especially promising for emotion research, as films evoke a higher intensity of emotion compared to other methods^7,8^. The ecological validity of the participants’ emotion experience is also superior when elicited by films, because events dynamically unfold over time and allow a natural evolution of emotions across successive moments. There is an increasing amount of publicly available fMRI datasets that include film watching (e.g.^3,9–11^), yet without annotations regarding emotion elicitation these have limited value for affective neuroscience. Acquiring such annotations is very resourceful as to date it is not possible to reliably extract rich information on experienced emotion in an automated fashion from audiovisual contents themselves, e.g., by relying upon artificial intelligence. Therefore, a large community effort is needed to produce and share film fMRI datasets that include detailed emotion annotations. Furthermore, the inclusion of detailed physiological measurements is needed to better understand the sources of the neural signal and the effects of emotional stimuli on emotion^12^. The StudyForrest initiative is a prominent example of an existing dataset including MRI and physiological recordings as well as annotations of portrayed emotions with the Forrest Gump movie being the central stimulus^10^. This initiative also shows how public datasets of this kind can be expanded upon with additional data acquisitions pertaining emotional content^13^. Similarly, previously annotated film stimuli can be utilized as stimuli in neuroimaging experiments to study emotion in the brain^14,15^.

While neuroscience research on emotion has long been dominated by bidimensional (valence and arousal) or core affect models^16^, appraisal theories are currently receiving increasing attention and already made important contributions in psychology^17,18^. These theories comprise a group of models predicting that emotions are determined by an individual’s appraisal of a current event or stimuli in relation to their goals and needs^18^. Although appraisals constitute a well-established mechanism of emotion elicitation, this framework was only rarely investigated in neuroimaging studies^19,20^. There is a need to better characterise the neural processes of cognitive appraisal and subsequent emotional responses, especially given the potential to inform our understanding of perturbed emotion processing in psychopathology by directly building on these insights.

Here we focus on a specific appraisal theory, the Component Process Model (CPM), initially proposed by Scherer^21^. The CPM describes how emotion is composed of a set of five distinct components: appraisal, motivation, expression, physiology, and subjective feeling. Compared to other frameworks, the CPM comes with a larger library of resources for empirical research. Most notably, the Geneva Emotion Recognition tool (GRID) instrument provides a collection of emotion words and features in accordance with the CPM and other theoretical approaches to emotion, such as the dimensional and the basic emotion approach^22^. A small number of fMRI studies have based their investigations on the CPM so far^19,23,24^; however, rating data (available to the wider community) did not include rich moment-by-moment annotations of film content but were based on preselected snapshots or experimentally induced^19,23,24^. Although our annotation measures were especially tuned (though not limited) to variables delineated under the theoretical framework of the Component Process Model^21^, these may generalise easily to other appraisal models and more generally be integrated with other common emotion theories.

We present the EmoFilm dataset, that was obtained by combining an annotation with a neuroimaging study. In the annotation study part, we use a selection of 16 short films for which have previously been annotated for valence and arousal^25^ and for aesthetic highlight^26^. We added to these existing data new continuous annotations for another 55 emotion-relevant items, 13 from the domain of discrete emotions and 42 from the categories of appraisal, motivation, expression, physiology, and subjective feeling based on the GRID instrument^22^. Based on ratings from 44 annotators, we calculated a consensus annotation to describe the general pattern of behavioural responses to the films’ content. In the neuroimaging study part, we collected fMRI and physiological data from an independent sample of 30 participants watching 14 of these short films where reliable consensus ratings could be calculated in the annotation study. We also included a behavioural task after fMRI scanning during which participants rated short clips taken from the same films in order to validate the continuous ratings obtained in the annotation study. The films used here are all in the public domain and thus easily available for researchers who wish to extract information from them or extend this dataset with their own acquisitions.

The multimodal data from both study parts, i.e., annotations of emotion experience and the corresponding physiological and fMRI recordings, can be put in relation to one another to investigate the effects of emotion experience during film watching in terms of various emotion descriptors. The primary purpose of our new dataset is to reduce the gap between theory in psychology and empirical neuroscience research on emotion, through a refined characterization of brain activity patterns and dynamics in relation to a broad range of emotion experiences. This dataset is tuned to understand the universal processed underlying emotion processes rather than studying related individual differences. In addition, we see many other opportunities offered by these data for a wide variety of research applications.

## Methods

### Annotation study

#### Participants

Forty-four participants (23 female) were recruited over the course of the study to perform film annotations remotely using their own computers. The mean age was 25.31 with a range from 20–39. Inclusion criteria were high oral comprehension level for English, no history of psychiatric or neurological diseases, no recreational drug use as well as no current neuropharmacological medication. Despite the online nature of data collection, we deliberately recruited participants locally, from Geneva university and the surrounding population, expecting a higher data quality with stronger motivation and better match with subsequent fMRI sample. Recruitment was performed via a questionnaire that was circulated online in relevant groups and forums within the university community and the wider population in the Geneva area. As some participants eventually failed to complete the whole experiment, we recruited four additional participants to compensate for missing data. Participants were reimbursed with 20 CHF/hour upon completion of the experiment. In total, forty-four participants completed the experiment between November 2020 and February 2021. One participant completed the experiment in January 2022 and another in October 2022, after they were recruited to replace corrupt or missing data. Ethical approval was given by the Geneva Cantonal Commission for Ethics in Research (protocol No 2018-02006). The study complied with the Code of Human Research Ethics (2014). All participants gave written informed consent prior to taking part in the study and were transparently informed of research goals.

#### Materials

##### Films

Emotion annotations were acquired for 16 short films taken from the films included in the LIRIS database^25^ and previously used for affective research. All selected films are free to share under Creative Commons licences. They were chosen based on their potential to evoke a broad range of emotions, but also based on logistical considerations, including film duration, diversity of content or format. For the purpose of this research, the beginning and end credits were cut. Our dataset includes the resulting clips ranged in duration from 6 minutes 42 seconds to 17 minutes and 8 seconds (average 11 minutes 47 seconds). Table 1 details the duration of each film with information on their genre and content.

**Table 1:** List of films used in the fMRI study, with film duration, scan duration, content description, film genre, and average ratings of absorption, enjoyment, and interest given by participants after scanning.

Film	Film duration (s)	Scan duration (TRs) - includes washout	Description	Genre	Absorption	Enjoyment	Interest
					Mean of ratings on a scale 0–100		
After The Rain	8:16	534	A man contemplates the meaning of life and human interaction on a rainy day in the city.	Drama	49	47	49
Between Viewings	13:28	776	A disillusioned estate agent is forced to re-examine his life when asked to sell his childhood home.	Comedy/ Drama	64	69	68
Big Buck Bunny	8:10	528	An enormous, fluffy, and adorable rabbit is harassed by a bullying gang of other forest animals.	Animation/ Comedy	74	78	68
Chatter	6:45	464	A girl witnesses a horrible sight online, then the electricity is cut off inside her apartment. When the light returns, she feels that she is not alone.	Thriller	69	50	60
Damaged Kung Fu*	15:22	n/a	Two friends are shooting a Kung Fu film together, when one quits thinking his friend is having an affair with his girl friend. The crew try to replace him in the shoot with little success.	Action/ Comedy	n/a	n/a	n/a
First Bite	9:59	613	A teenage girl is discovering the power of seduction.	Romance	55	51	52
Lesson Learned	11:07	665	A young man turns his life around after being involved in gang violence.	Drama	60	53	61
Payload	16:48	928	A man must sacrifice everything to save his family.	Drama/ Sci-Fi	55	53	60
Riding the Rails*	13:14	n/a	A boy’s grandfather tries to cheer him up on his birthday with a new train for his train set.	Drama	n/a	n/a	n/a
Sintel	12:02	710	A young woman embarks on a dangerous quest to find her lost friend, a dragon.	Animation/ Fantasy	85	80	83
Spaceman	13:25	744	A young man sets out on a very curious and unique path to realise his dream of being an astronaut.	Romance/ Drama	60	65	68
Superhero	17:08	945	A single mother cares for her son with terminal cancer.	Drama	65	56	60
Tears Of Steel	9:48	607	A group of soldiers and scientists try to stop an army of robots that threatens the planet by correcting a past mistake.	Action/ Sci-Fi	68	69	71
The Secret Number	13:04	757	A psychiatrist is compelled by his patient, an obsessive mathematician, to consider the existence of a secret integer between three and four.	Drama	77	80	81
To Claire From Sonny	6:42	460	A young man writes a letter to his first true love.	Drama	71	73	73
You Again	13:18	768	A chance encounter between two former high school sweethearts forces them to face the ways they have - and have not - changed.	Romance/ Drama	45	49	46

*indicates films that were not included in the fMRI acquisition

##### Annotation items

In our study, 55 items were annotated comprising 42 items from the categories of Appraisal, Expression, Physiology, Motivation and Feeling that were adapted from the CoreGRID instrument^22^, plus a further 13 terms for discrete emotions (see Supplementary Table 1 for a list and description of all items).

##### Questionnaires

A number of questionnaires were given upon completion of the emotion annotation task. We used total scores computed from these questionnaires. The Depression Anxiety Stress Scales (DASS^27^) was used to assess affective state over the previous seven days. We also employed an in-house scale to gauge how people were affected by the Covid-19 pandemic and its consequences. The scale includes items rating the pandemics’ effect on social support, mental health, concerns about getting infected or infecting others, worries about the future and impact on cognitive function (internal consistency; alpha = 0.80). This scale has not been validated. The BIS/BAS Scale^28^ was used to measure the behavioural approach system on the subscales drive, fun seeking, and reward responsiveness, and the behavioural inhibition system. We also administered the Emotion Regulation Questionnaire (ERQ^29^), which probes two facets of emotion regulation: Cognitive Reappraisal and Expressive Suppression, as well as the Big Five Inventory (BFI^30^) which was used to create scores of Extraversion, Agreeableness, Conscientiousness, Openness, and Neuroticism. Description of the sample based on these questionnaires can be found in Supplementary table 2. Responses were not available for two subjects.

#### Procedure

##### Annotation tasks preparation

Annotation tasks were generated as a ‘to-do-list’ for participants before recruitment. These annotation tasks took the form of.mp4 files of the films named following a specific format so that both the participants and the annotation software could recognize the task.

In order to generate four annotations for all 16 times 55 films by item combinations we randomly assigned six items to each of 37 initial lists of annotation tasks (as participants did not complete the full list of tasks the final number of annotators used is 44). Thus, each participant’s annotation tasks were comprised of 16 times six film by item combinations (=96 tasks), sorted in blocks by item. This means that participants would annotate one item for all 16 films in random order, before moving on to the next item. We do not expect adverse effects due to participants viewing each film multiple times based on the relatively long delay between repetitions and findings that repeating a specific emotional stimulus has only a negligible effect on self-reported emotional feelings^31^. Each item was assigned to four annotators, thereby allowing us to assess agreement between annotators and calculate a consensus annotation later on. Supplementary Table 3 shows in a binary grid format which film by item combinations were rated by which annotator.

Along with the annotation task lists we prepared detailed instructions pertaining to the interpretation and directionality of the items to ensure uniform interpretations across our sample. All materials were accessed by participants via a dedicated online platform.

##### Annotation software

To obtain online ratings, we used an adapted version of the software CARMA^32^, specifically developed for film annotations. The main customizations were related to the annotation scale adapting to the current item, which was specified in the file name of each rated film, and naming outputs including film, item and participant names. The sampling rate within CARMA software was fixed to 1 Hz. To complete their annotation tasks, participants would load the prepared video files according to the order in their annotation task list and then move a mouse-controlled cursor along a unitless bar on the computer screen to continuously annotate an item. A short verbal descriptor was displayed on the upper and lower poles of the scale as in Supplementary Table 1. Data was recorded on a scale from 0–100.

##### Film annotation

Upon recruitment, participants were contacted with detailed information about the study and invited for a video call with a researcher. During this meeting, participants were instructed how to download and use the annotation software (CARMA^32^), how to access their annotation tasks and item descriptions, and how to upload completed annotations onto a secure online platform. A researcher further explained in detail how assigned items should be interpreted and which directionality they should be rated in. In addition, participants were able to access brief descriptions of the items in written form, such that they could consult them when needed.

Participants were instructed to complete the list of annotation tasks in a given order at their own pace. They were encouraged to complete all annotations within six weeks. Upon completion of a session, they were instructed to upload their response files onto the secure online platform. This ensured that the quality of their annotations could be monitored continuously.

##### Continuous quality control

Continuous quality control was performed using visual inspection of time-courses and analysis of agreement between raters when applicable. Participants received feedback if their time courses appeared too “synthetic” (e.g., box-shaped or constant) or if there was an unexpectedly high discrepancy between their annotations and the rest of the cohort. No participants were excluded based on annotation quality.

#### Calculation of consensus annotation

All annotation time series were z-scored across films within each rater before further processing. Individual missing values in time series were replaced with the mean of the two neighbouring values (a total of nine values were replaced in this way). Constant time series were discarded and not included in the calculation of the consensus annotation, as were time series with outliers beyond a z value of 15 or −15. Finally, the quality of annotations was assessed using Pearson’s correlation coefficient (r). Specifically, for each item and each film, r was calculated between each pair of annotations across participants (resulting in 6 r values), and then averaged across pairs to result in one value of agreement per film and item. If the inclusion of a time series reduced mean r between all raters by more than .20, the time series was discarded for calculation of a consensus annotation (except for four cases where this would have left only two time series to calculate the consensus). Finally, in a few exceptional cases where five annotations were available (due to additional recruitment, see above), we removed the annotation with the lowest average correlation with the other annotations. The remaining complete time series were averaged per item and per film to form the consensus annotation. Each consensus annotation time series was based on the average of at least three raters.

### fMRI study

#### Participants

Thirty-two healthy volunteers were recruited for the fMRI experiment. Two had to be excluded during the first session, because one could not tolerate lying in the MRI scanner and one had strong artefacts due to dental braces. Consequently, 30 subjects (18 female) completed the fMRI experiment, none of which partook in the annotation study. All subjects were healthy adults between 18 and 35 years old (average 25.83, std = 3.60) and right-handed as confirmed with the Edinburgh Inventory^33^. All had normal or corrected-to-normal vision including full colour vision, high level of English language comprehension, no history of any neurological or psychiatric condition, and none reported using neuropharmacological or recreational drugs. Ethical approval was given by the Geneva Cantonal Commission for Ethics in Research (protocol No 2018-02006). The study complied with the Code of Human Research Ethics (2014). All participants gave written informed consent prior to taking part in the study and were transparently informed of the research goals at all times.

#### Materials

##### Films

We used 14 short films selected from the previous annotation study (see Table 1). Two films were not included because of unreliable consensus annotations (*Damaged Kung Fu* and *Riding the Rails*). We used the same clips as before, without beginning and end credits. The average duration of these films was 11 minutes 26 seconds.

##### Annotation Items

After scanning, we included an offline behavioural rating phase to validate the annotations obtained from other participants in the annotation study. We used a subset of 48 items comprising 34 items from the categories of Appraisal, Expression, Physiology, Motivation and Feeling taken from the CoreGRID^22^ and 13 discrete emotion terms. We did not include items for which we found no reliable consensus annotation in the annotation study (see Supplementary Table 1 for list and description of all items).

##### Questionnaires

We used the same battery of questionnaires as in the annotation study. A description of the sample based on these questionnaires is provided in Supplementary Table 2.

#### Procedure

##### Imaging experiment

The experiment spanned over four fMRI sessions each lasting approximately two hours. During these sessions, subjects watched between two and five short films in the MRI scanner and subsequently rated their emotion experience during watching in the offline behavioural test. Additionally, subjects underwent a 10-minute resting-state scan in the first session during which they were asked to keep their eyes open and fixate a crosshair on the screen. Each subject watched the films in pseudo-random order, distributed over the four sessions. Stimulus presentation was programmed in Matlab 2012, using the Psychophysics Toolbox extensions^34–36^. This program also recorded stimulus onsets and offsets for each film and rest blocks. Each film run started and ended with a 90 second washout period during which a crosshair was presented centrally on the screen without auditory stimulation. Between the two washouts, the film was displayed on the screen with the corresponding audio track heard through in-ear plugs. The subjects were instructed to watch the films as they would watch films in their everyday life. At the end of each film run, participants responded to three successive questions, displayed in white on a black background on the screen, to indicate their level of absorption (‘I felt absorbed by this movie’), enjoyment (‘I enjoyed this movie’), and interest (‘I thought this movie was interesting’) during film watching. They used a button box to move a slider on the screen up or down a continuous unitless scale to mark their agreement with the respective statement. Values were recorded on a scale from 0 to 100.

##### MRI data acquisition

MRI scans were acquired on a 3 T Siemens Magnetom TIM Trio scanner (Siemens, Erlangen, Germany) using a 32-channel head coil at the Brain and Behaviour Laboratory at the University of Geneva (BBL). Structural T1 weighted images, used for co-registration, were acquired with a standard Siemens MPRAGE sequence (TR = 1.9 s, TE = 2.27 ms, TI = 0.9 s, flip angle = 9°, GRAPPA = 2, 24 reference lines, 192 slices, FoV read = 256 mm, voxel size = 1 × 1 × 1 mm³, sagittal orientation, PE = AP, no fat or water suppression, single shot MB mode, bandwidth = 190 Hz/Px, Echo spacing = 6.7 ms, TA = 304 s). All functional images were acquired with the same simultaneous multi-slice (a.k.a. multiband, MB) gradient-echo planar imaging sequence provided by the Centre for Magnetic Resonance Research (CMRR, Minnesota)^37,38^ (TR = 1.3 s, TE = 30 ms, flip angle = 64°, MB acceleration factor = 3, interleaved MB mode, 54 slices, FoV read = 210 mm, voxel size = 2.5 × 2.5 × 2.5 mm³, PE = AP, bandwidth = 2290 Hz/Px, Echo Spacing = 0.57 ms, EPI factor = 84, Pulse duration = 4300us, fat saturation). Resting-state runs lasted 10 minutes, totalling 460 volumes. The number of volumes acquired for each film and the duration of each film are detailed in Table 1. Slice timing for each scan can be found in the corresponding sidecar file in the BIDS dataset.

##### Physiology acquisition

Participants’ physiological activity was recorded for the whole duration of each fMRI scan with a BIOPAC MP150 monitoring system and recorded with the AcqKnowledge software (version 4.4). Specifically cardiac pulse was collected via photoplethysmogram (BIOPAC TSD200_MRI transducer and PPG100C amplifier), respiratory effort was measured via chest expansion (BIOPAC TSD221-MRI fully pneumatic respiration transducer and RSP100C amplifier), and skin conductance was collected via Electrodermal Activity (EDA) (Cleartrace electrodes 2 RTL and EDA100C amplifier). All signals were sampled at a rate of 1000 Hz. Physiological recordings encompassed the whole acquisition.

##### Validation of film annotations

Once outside the MRI scanner, participants completed an offline behavioural task where they rated their emotion experience during the films, they had just seen during the fMRI session. This task was programmed in Matlab 2012 using the Psychophysics Toolbox extensions^34–36^. Participants were given instructions pertaining to the meaning and directionality of the rating items to ensure adequate understanding and uniform interpretations across our sample. During this task, they re-watched selected clips from each film (on average ~21 clips per film) and rated them by moving a slider up and down along a continuous scale (without units or markers) whose extremities indicated high and low experience. Values were recorded on a scale from 0 to 100.

Participants rated five different items sequentially after seeing a short clip. In total, each participant watched and rated 292 clips taken from the films, with an average duration of 7 s, equating to 20.52% of the total duration of all films (range = 14.79%–26.37% of each film). Each item was rated by three to four subjects.

#### Data processing

##### Physiology preprocessing

AcqKnowledge proprietary files containing physiological data were organised into the Brain Imaging Data Structure (BIDS)^39^ schema with phys2bids^40^. The conversion process simultaneously splits files into runs, keeping extra recording material before and after the run itself (9 s on both sides), and converting them into tabular (tsv) format.

After downsampling both cardiac pulse and ventilation data to 40 Hz and applying a low-pass filter (8 Hz for cardiac data and 2 Hz for ventilation), peaks were detected automatically, with manual supervision, using peakdet^41^. The denoised physiological data was used to model physiological noise with phys2denoise^42^, in the form of Heartbeat Interval (HBI) and Respiratory Variance (RV). Briefly, HBI was computed as the median of peak-to-peak distance within a sliding window of 6 s, convolved with the opposite of the cardiac response function^43^. RV was computed as the variance of the signal within a sliding window of 8 s, convolved with the respiratory response function^44^.

##### fMRI preprocessing

MRI DICOM files were organised following the BIDS schema with BIDScoin^45^, and simultaneously converted to nifti with dcm2niix^46^. fMRI data processing was conducted using FEAT (FMRI Expert Analysis Tool) Version 6.00, part of FSL (FMRIB’s Software Library, www.fmrib.ox.ac.uk/fsl). Images were coregistered to a high-resolution structural, standard space and to the first functional volume of each subject using FLIRT^47,48^. The following preprocessing pipeline was applied; motion correction using MCFLIRT^48^, non-brain removal using BET^49^, spatial smoothing using a Gaussian kernel of FWHM 6.0 mm; grand-mean intensity normalisation of the entire 4D dataset by a single multiplicative factor; high pass temporal filtering (Gaussian-weighted least-squares straight line fitting, with sigma = 50 s). We further used FAST segmentation^50^ to identify tissue classes at subject level and regress average time courses from white matter (WM) and cerebrospinal fluid (CSF) from the data together with the six motion regressors derived from image realignment. Finally, we applied defacing to the structural images using pydeface (v. 2.0.0)^42^.

##### Calculation of agreement between consensus annotation and validation

To compare the clip ratings in the validation task with the continuous annotations from the annotation study, we applied a linear interpolation over ratings from the clips, then z-scored each time series, and computed an average over all subjects who rated the respective item. This average time series was then compared to the consensus annotation from the annotation study using Pearson correlation. We also compared the mean inter-rater agreement for each item to the mean correlation between the time courses from the fMRI study and the consensus annotation for each item derived from the annotation study.

## Data Records

The data for both the annotation and the neuroimaging parts of our study have been organised in BIDS format^39^ and can be both be found on OpenNeuro under https://openneuro.org/datasets/ds004872^51^ and https://openneuro.org/datasets/ds004892^52^ respectively. All data has been anonymised, including defacing of MRI scans.

## Technical Validation

### Annotation quality

Annotation quality was also assessed for each item and each film pairing using Pearson correlation. Agreement was strongly dependent on the item that was annotated with the highest agreement being r = 0.58 for *PleasantOther* and the lowest being r = −0.01 for *Jaw*. Mean agreement also differed between films (ranging from 0.21–0.49). While it is natural for agreement to vary between items and films, we recommend that items with mean agreement of smaller r = 0.20 across films cannot be considered reliable. Consequently, the following items were removed from all further analysis as their agreement across all films was smaller than r = 0.15: *Breathing* (r = 0.14), *Consequences* (r = 0.11), *Movement* (r = 0.07), *EyesOpen* (r = 0.06), *Jaw* (−0.01). We also removed two films that did not achieve a mean agreement of at least 0.25 across all items: *Riding the Rails* (r = 0.21) and *Damaged Kung Fu* (r = 0.24).

From the remaining 14 films and 50 items, 2840 individual annotation time series were available. Of these, two annotation time series were removed because of constant segments, and 48 because of outliers beyond a z-value of 15 or −15. In addition, 126 time series were removed as being deviant (their exclusion improved mean inter-rater agreement by more than r = 0.2) and 18 annotations were removed as they were the worst of five in terms of inter-rater agreement.

**Fig. 1:**
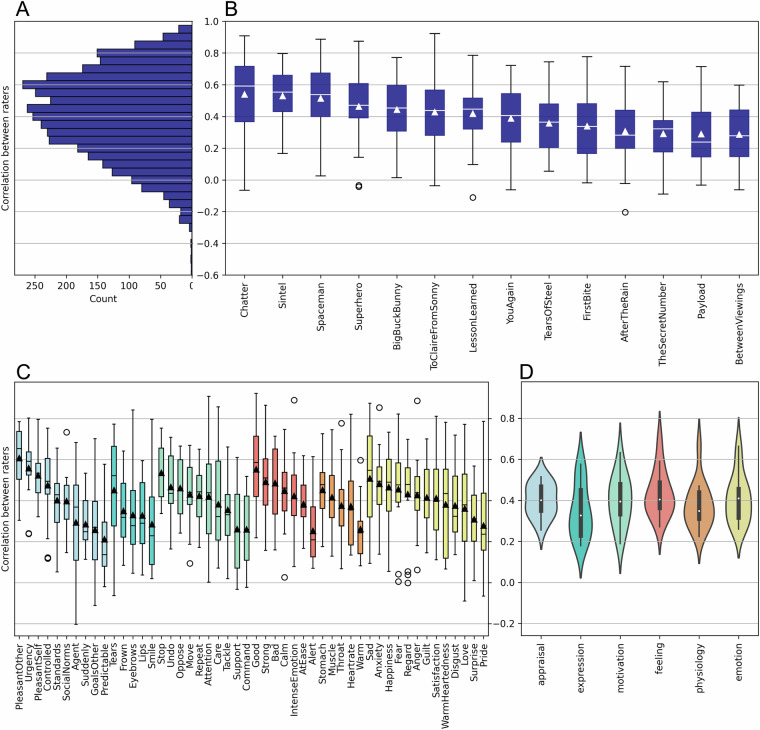
Inter-rater agreement in annotation study. (**A**) Histogram of inter-rater agreement between all valid pairs of ratings. (**B**) Distribution of inter-rater agreement by film based on all valid pairs of ratings. (**C**) Distribution of inter-rater agreement by item based on all valid pairs of ratings, including GRID items and discrete emotion terms. Bars are coloured by components according to the CPM framework, as in (**D**). (**D**) Distribution of inter-rater agreement by components and discrete emotions.

Consequently, Fig. 1 shows a detailed summary of agreement overall (A), within films (B), and across rating items (C) and CPM categories (D), after removal of the two films and five items with poor reliability. The final dataset therefore includes annotations from 50 items for 14 films, with an average agreement for films across items between r = 0.29–0.54, and agreement for items across films between r = 0.21–0.60. Of the 700 film and item combinations in these data, the final consensus annotation was calculated based on three annotations for 154 cases, and based on four annotations for all remaining ones. The mean agreement across all items and films was r = 0.39 (see Supplementary Table 4 for detailed Inter-rater agreement across all films and items).

**Fig. 2:**
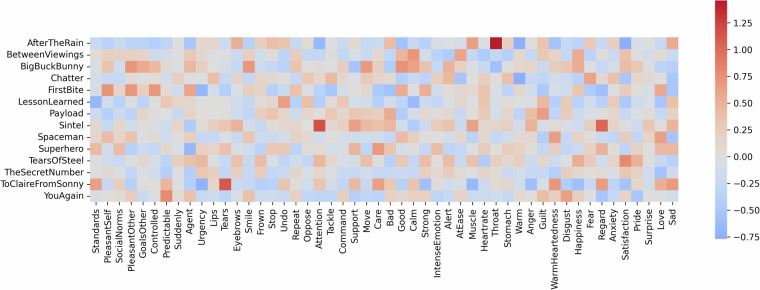
Average value of consensus annotation for each item and film. High values (red) indicate films that were rated consistently higher on this item relative to other films. Conversely, low values (blue) indicate films that were rated consistently lower on this item relative to other films.

We furthermore report the average value from the consensus annotation for each item in each film in Fig. 2. This illustrates the expected variety of relative emotion intensity between films, but also shows that various emotion dimensions were generally elicited within a given film.

### Validation of annotations

**Fig. 3:**
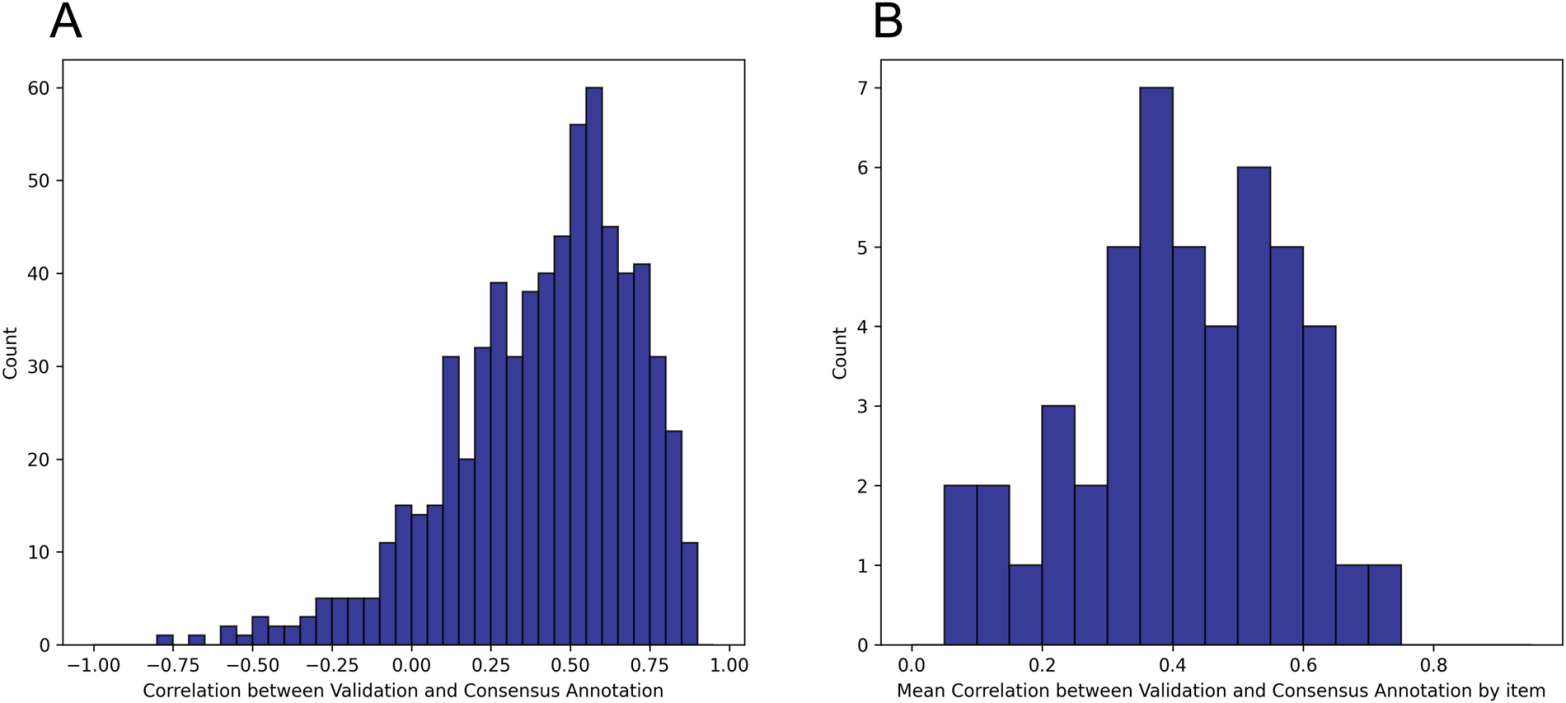
Histograms of the correlation values between validation time courses and the consensus annotation. (**A**) Agreement for all combinations between films and items and (**B**) for the average value within each item across films.

The validity of ratings acquired in the fMRI study was verified by comparing them to the consensus annotation obtained in the preceding annotation study part by computing Pearson correlations between the average ratings from the fMRI study and the consensus annotation from the annotation study. The mean correlation across all films and items was 0.41. This is comparable with the mean inter-rater agreement reported previously. Figure 3 shows histograms of the correlation values between the validation time courses and the consensus annotation, for all combinations between films and items (A) and for the average value within each item (B). The mean agreement with the consensus annotation ranged from 0.08 for *Regard* to 0.70 for *Stop*., but with clear peaks between 0.4 and 0.6.

For most items, the agreement with the consensus annotation was higher than the inter-rater agreement within individual ratings in the annotation study. The correlation between the mean inter-rater agreement for each item in the annotation study and the mean correlation of the validation time course with the consensus annotation was .63. This means that items that reached lower agreement in the annotation study also showed lower agreement between the consensus annotation and the validation time series. This may be a feature of these items, i.e., they may not be experienced as universally as others and potentially depend more on individual differences; or a specificity of the current film material, i.e., some items were not appropriately evoked by the content of selected films.

### fMRI quality control

**Fig. 4:**
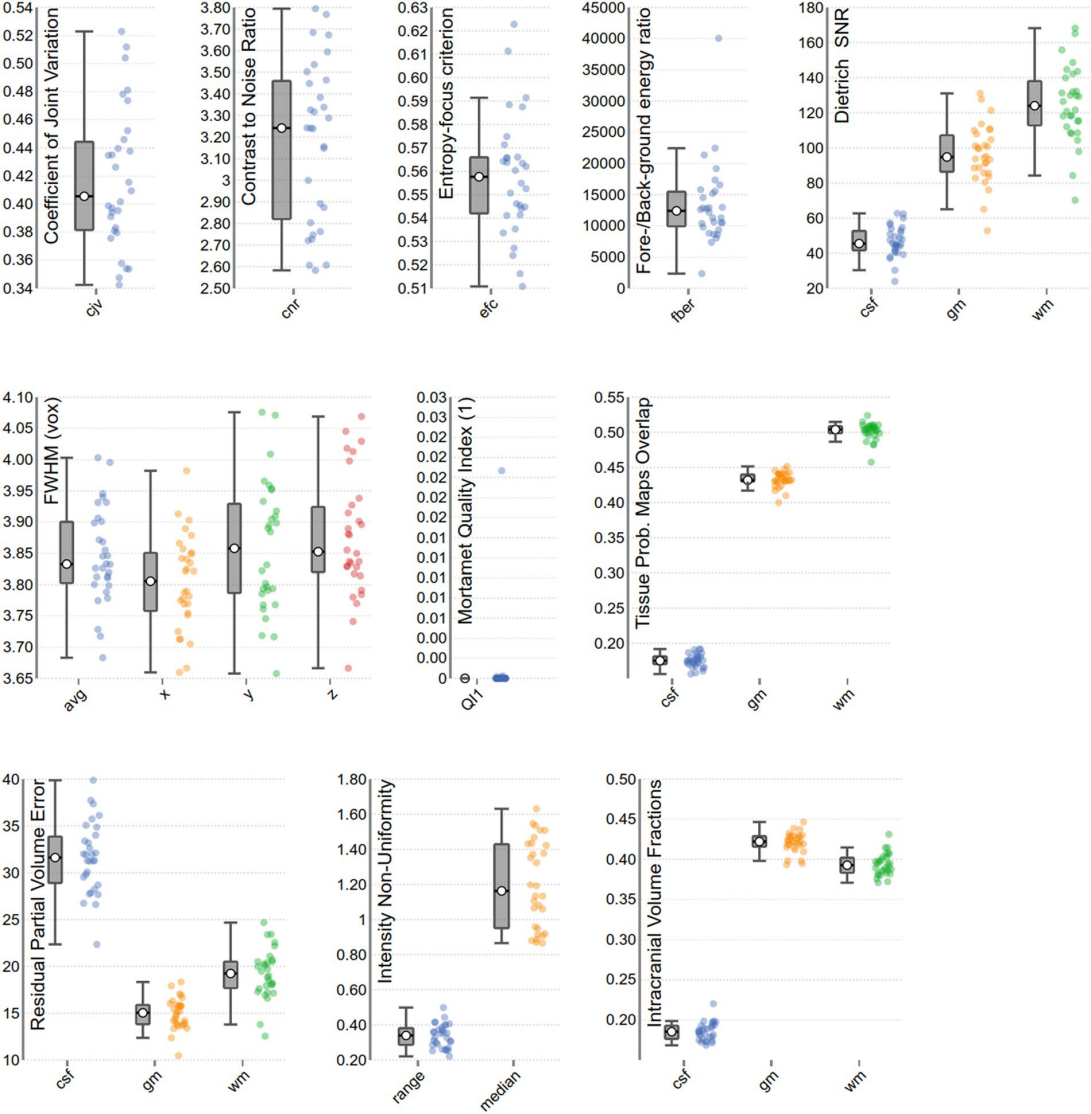
Subgroup of quality metrics of T1w volumes, computed by MRIQC. Each dot represents a volume. For an in-depth explanation of each metric, see^61^.

**Fig. 5:**
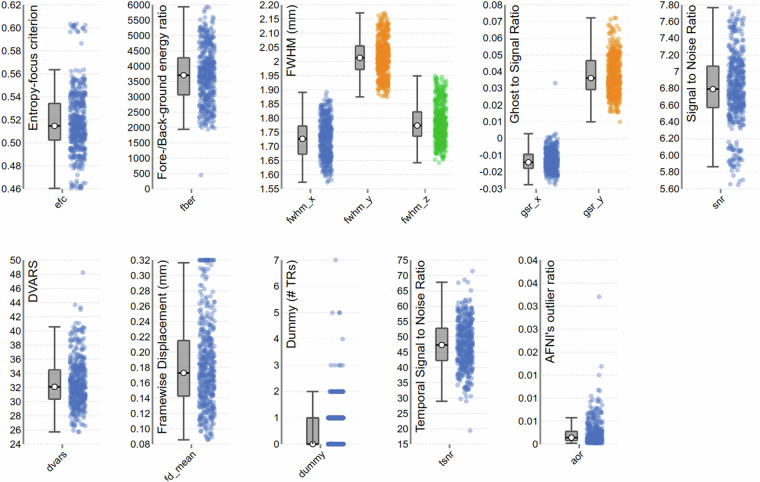
Subgroup of quality metrics of BOLD runs computed by MRIQC. Each dot represents a run. For an in-depth explanation of each metric, see^61^.

MRIQC (v. 0.16.1)^53^ was used to assess quality control of both structural and functional MRI data. Figures 4, 5 report a subset of the quality metrics computed by MRIQC, for structural and functional volumes respectively. The integral reports can be found in the derivatives of the fMRI dataset on OpenNeuro^52^ (10.18112/openneuro.ds004892).

For the structural images, the coefficient of joint variation^54^ indicates absence of heavy head motion, as does framewise displacement (FD)^55^ although the intensity non-uniformity index^44^ indicates sub-optimal field bias. There seems to be low ghosting and blurring induced by head motion, with few volumes showing an entropy-focus criterion (EFC) higher than 0.58^56^. The contrast to noise ratio and Dietrich Signal to Noise Ratio (SNR) (a comparison between tissues and background, see^57^ are high, especially for Grey Matter (GM) and White Matter (WM), although the average image smoothing median is 3.83 voxels. Mortamet’s Quality Index^58^ indicates no voxels with intensity corrupted by artefacts, with the sole exception of a few voxels in the anatomical volume of subject 16.

Most functional volumes show an EFC below 0.57, with the exception of the runs of subject 29, and a film run from subject 20 (The Secret Number). Data smoothness is within the voxel size, the Ghost to Signal Ratio close to 0, although higher in the phase-encoding axis y, and SNR and temporal SNR (measure of MRI signal strength) are high, with very few outliers found by AFNI’s 3dToutcount^59^, beside subject 21 rest run, overall indicating acceptable data quality. The number of initial volumes labelled as “dummy”, due to non-steady magnetisation state is within 2 volumes for most runs, with a few exceptions.

Regarding functional runs FD, across all functional runs mean FD was 0.16 mm (SD = 0.10) ranging from 0.09–0.55 mm. While FD was generally low, we found a significant difference of FD between film (M = 0.17, SD = 0.10) and rest (M = 0.12, SD = 0.04), with rest having significantly smaller FD (t(448) = 2.72, p < 0.01). This is contrary to previous findings of reduced motion during film fMRI compared to rest^60^. No subjects were excluded based on MRI image quality.

We furthermore conducted a visual validation of stimulus onsets and offsets during the film runs. For this purpose, we plotted regional time courses of preprocessed BOLD signal from the visual cortex for all subjects centred on the recorded film onset. This demonstrated an increase in signal as well as synchronization between subjects’ time courses during film blocks. The detailed results from this analysis can be found in the derivatives of the fMRI dataset on OpenNeuro.

## Supplementary information

Supplementary Tables

## Data Availability

We used standard processing pipelines for most of our data and all software used for fMRI processing is freely available for researchers (e.g., www.fmrib.ox.ac.uk/fsl). Some custom scripts were used and have been made available on github (https://github.com/EllieMo/Emo-FilM).
